# Optimization of Pulley-Type Ring Resonator with Waveguide Offset

**DOI:** 10.3390/mi9050226

**Published:** 2018-05-10

**Authors:** Meng-Hua Yen, Pei-Yu Feng, Chu-En Lin, Chii-Chang Chen, Jenq-Yang Chang

**Affiliations:** 1 Department of Mechanical Engineering, National Chin-Yi University of Technology, Taichung 411, Taiwan; emh1989@mail.ncut.edu.tw (M.-H.Y.); celin@ncut.edu.tw (C.-E.L.); 2 Department of Optics and Photonics, National Central University, Taoyuan 320, Taiwan; 102226020@cc.ncu.edu.tw (P.-Y.F.); jychang@dop.ncu.edu.tw (J.-Y.C.)

**Keywords:** ring resonators, waveguide offset, Q-factor

## Abstract

In this work, we dealt with the optimization of the pulley-type ring resonator using the offset of the straight input and output waveguide at the junction with the curved waveguide. We adopted the finite-difference time-domain method to simulate the structure. It was found that the coupling loss could be significantly reduced and the critical coupling could be precisely tuned. This results in the possibility of the Q-factor being higher than that of the structure without waveguide offset. In this study, the Q-factor of the ring resonator is increased from 9180 to 11,302. The corresponding enhancement is 23.1%.

## 1. Introduction

The ring resonator based on silicon-on-insulator (SOI) technology is an important and versatile integrated optics component. Ring resonators have been well-studied in the past decade and have been widely applied for purposes such as optical filters [1,2], optical switches [3,4], optical modulators [5], ring lasers [6,7], biomedical photo-detectors [8,9,10], displacement detectors [11], ultrasonic sensors [12], chemical sensors [13], stress and strain sensors [14], polarization filters [15,16] and slow light [17]. All of these applications require a high quality factor—Q-factor. In recent years, several types of ring resonators have been developed, such as single ring resonators with a bus waveguide [18,19], racetrack ring resonators [20], multiple rings [21], and pulley-type ring [22,23]. Recently, we reported the procedure for designing a pulley-type ring resonator (PTRR) [23] with a high Q-factor. By tuning the waveguide width, a high-performance ring resonator could be easily obtained.

To reduce the waveguide loss, waveguide offset has been proposed as one of the solutions [24,25,26]. Subramaniam et al. have reported the bending loss and transition loss at S- or U-type channel waveguides with waveguide offset [24]. Howley et al. have also reported that the structure of the junction offsets is effective at reducing the bending loss of low-index contrast polymer waveguides [25]. Gu et al. have proposed a photon-hopping theory in which the received energy intensity at the end of waveguide offset can be enhanced by more than 50% [26]. The mode coupling between the abrupt change in the waveguide structure can be optimized by using this technique. In this study, the waveguide offset is applied to reduce the optical loss of the PTRR.

## 2. Structure and Simulation Results

**Figure 1:**
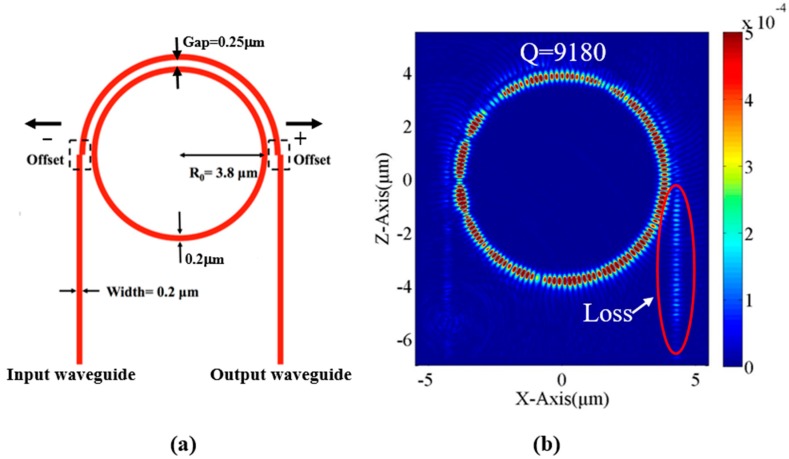
(**a**) Schematic of the PTRR. The symbols “+” and “−“ indicate that the straight input and output waveguides are shifted to the right to the left, respectively; (**b**) The distribution of optical field in the PTRR without waveguide offset.

The Q-factor is defined by the central wavelength of the resonant peak in the transmission spectrum of the ring resonator divided by the full-width at half-maximum of the resonant peak. To improve the Q-factor of the PTRR, the maintenance of light in the ring (i.e., reduction of the loss) is required. The schematic of PTRR studied in this work is shown in Figure 1a. The refractive index of the waveguide is 2.93. The refractive index of the surrounding material is unity. The radius of the ring is 3.8 μm. The waveguide width is 0.2 μm, which is determined in our previous experimental work to ensure the single-mode operation and to obtain a Q-factor as high as 173,000 [22]. The gap between the curved waveguide and the ring is 0.25 μm. Commercial software of the 2-D finite-difference time-domain (FDTD) method is used to simulate the structure. After the optimization procedure reported in Ref. [23], the light at the resonant wavelength of 1602 nm is launched into the input waveguide. The magnetic field of the input light is perpendicular to the propagation plane. The launch of the input light is stopped after the output intensity reaches stability. The optical field distribution of the PTRR is shown in Figure 1b. We can observe that the light is confined in the ring. A slight loss at the output waveguide is the main loss of the device. Therefore, the loss reduction at the output port may improve the Q-factor of the PTRR.

Since the light coupling between the curved waveguide and the straight input/output waveguide for the PTRR depends strongly on the waveguide geometry at the junction, in this study, we propose shifting the straight input/output waveguides to precisely tune the performance of the PTRR. Figure 1a also illustrates the offset of the straight input and output waveguides at the junctions with the curved waveguide. The shift of the straight input and output waveguides to the right and to the left is defined to be positive and negative offset, respectively.

**Figure 2:**
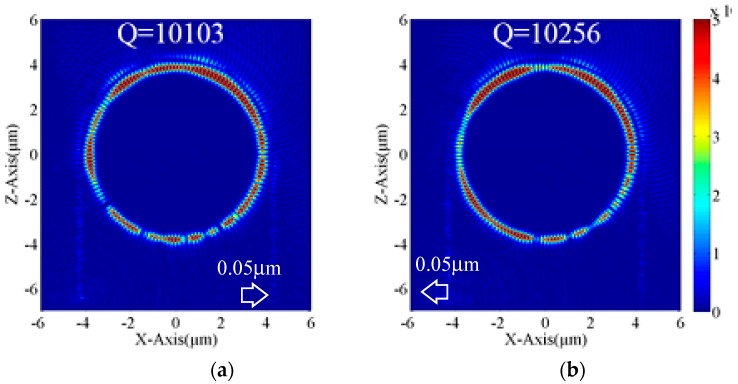
Optical field distributions of the PTRR with waveguide offset. (**a**) The output waveguide is shifted to the right by 0.05 μm; (**b**) The input waveguide is shifted to the left by 0.05 μm.

Figure 2 shows the optical field distribution of the PTRR with the waveguide offset. In Figure 2a, the straight output waveguide is shifted to the right for 0.05 μm. A significant reduction of the loss at the output waveguide can be observed. The Q-factor for the PTRR is 10,103 which is higher than that of Figure 1b. A Q-factor enhancement of 10% is achieved.

We also shift the straight input waveguide to tune the performance of the PTRR. In Figure 2b, the offset of the input waveguide and the output waveguide is 0.05 μm to the left and zero, respectively. The Q-factor of the PTRR may also be increased to 10,256.

**Figure 3:**
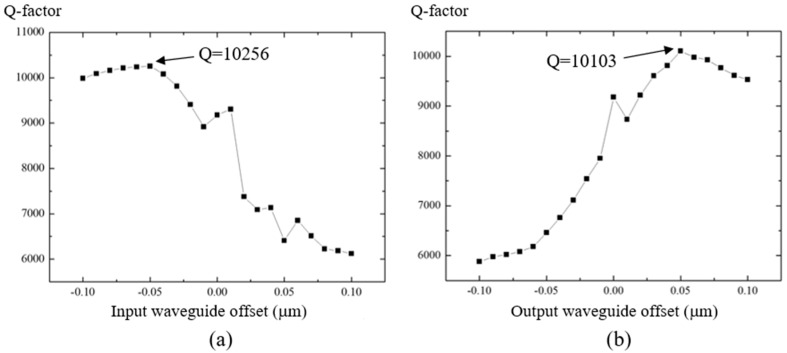

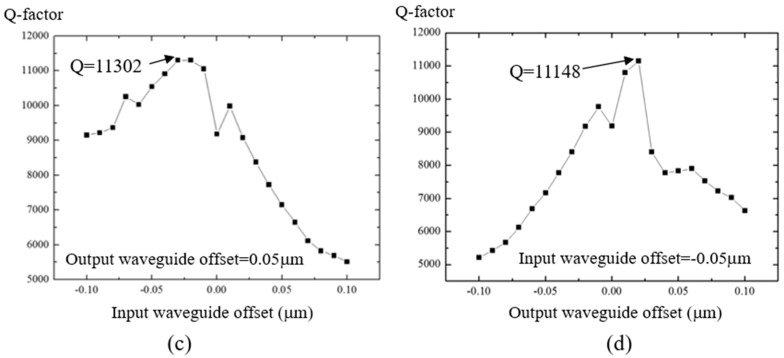
Q-factor for shifting (**a**) the straight input waveguide; (**b**) the straight output waveguide; (**c**) the straight input waveguide with offset of the output waveguide of +0.05 μm; and (**d**) the straight output waveguide with offset of the input waveguide of −0.05 μm.

Figure 3 shows the Q-factor variations for different waveguide offsets. The step of the waveguide offset is 0.01 μm, which can be realized by e-beam lithography in the fabrication process. By shifting the straight input waveguide, the maximum Q-factor was found to be 10,256, when the shift was −0.05 μm (Figure 3a). By shifting the straight output waveguide, the maximum Q-factor was found to be 10,103, when the shift was +0.05 μm (Figure 3b). We shifted the straight input waveguide in the condition of keeping the straight output waveguide with offset of +0.05 μm. The maximum value of the Q-factor was 11,302 when the offset of the straight input waveguide was −0.03 μm (Figure 3c). In the case of shifting the straight output waveguide while maintaining the straight input waveguide with an offset of −0.05 μm, the maximum value of Q-factor was 11,148 when the offset of the straight output waveguide was +0.02 μm (Figure 3d). The corresponding maximum enhancement of Q-factor of the PTRR for Figure 3c,d was 23.1% and 21.4%, respectively.

## 3. Optical Loss Analysis

**Figure 4:**
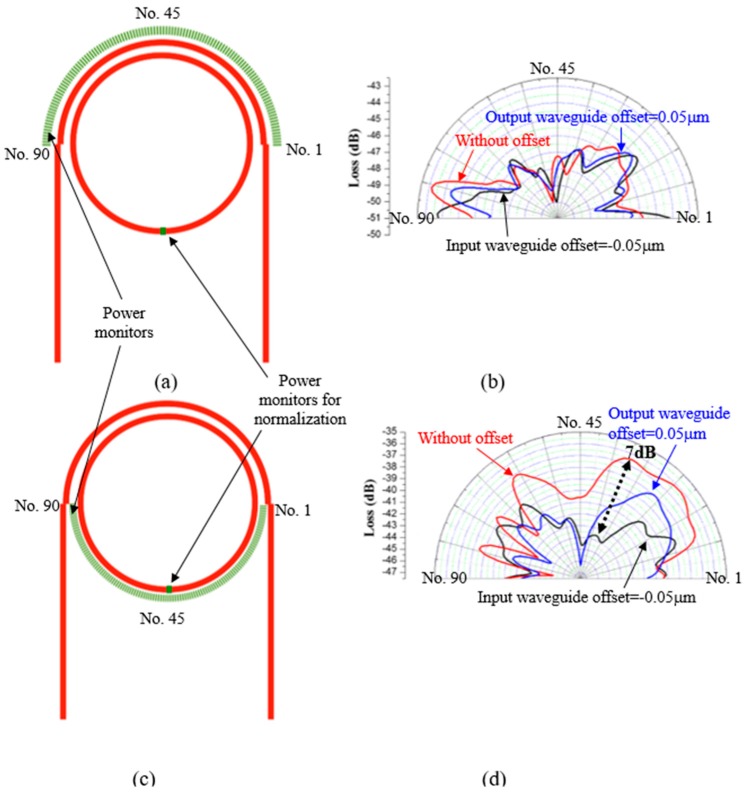
Position and the detected power distribution (**a**,**b**) for 90 power monitors outside of the curved waveguide, and (**c**,**d**) for 90 power monitors outside of the ring. The power detected by the 180 power monitors is divided by that of the power monitor positioned on the ring to obtain the normalized optical loss.

To analyze the optical loss of the PTRR with and without waveguide offset, the power monitors are placed outside of the ring resonator. Figure 4a shows the position of the 90 power monitors outside of the curved waveguide (upper half of ring). The distance between the power monitors and the curved waveguide and the ring is 0.4 μm. The power acquired from the 90 power monitors is divided by that of the power monitor on the ring to obtain the normalized optical loss. Figure 4b shows the optical loss of the curved waveguide of the PTRR with and without waveguide offset. We can observe that the optical loss is not obviously changed by the waveguide offset. In Figure 4c, the position of the 90 power monitors outside of the ring (lower half of ring) are illustrated. Figure 4d shows the optical loss of the ring of the PTRR with and without waveguide offset. We can observe that the optical loss of the lower half of the ring is significantly reduced by waveguide offset. The maximum reduction of the optical loss is around 7 dB. This indicates that mode coupling of the light at the junction of the straight and curved waveguides is ameliorated by the finely tuned waveguide offset. The results show that the waveguide offset can efficiently tune the performance of the PTRR to enhance the Q-factor.

The optical field remained in the input waveguide of Figure 1b and Figure 2a,b indicates the reflection of the light by the ring structure. We can observe that the optical field in the input waveguide is not significantly changed with waveguide offset, indicating that the reflection optical loss is not influenced by the waveguide offset.

## 4. Conclusions

In this work, the performance of the PTRR is tuned by shifting the input and output waveguides at the junction of the straight and curved waveguides. The light coupling at the junction and the optical loss of the ring can be significantly ameliorated. The Q-factor of the PTRR in this study can be improved from 9180 to 11,302. An enhancement of Q-factor of 23.1% is achieved. Many parameters of the ring resonator might be tuned to obtain a higher Q-factor. This work shows that the technique of the waveguide offset could be used as the loss at the junction of the straight waveguide and the ring is the main origin of the loss. This technique could be used to finely tune the ring resonator in which the Q-factor is much higher than that in this work.
